# Umbilical Cord Mesenchymal Stem Cell-Derived Extracellular Vesicles as Natural Nanocarriers in the Treatment of Nephrotoxic Injury In Vitro

**DOI:** 10.3390/cells13191658

**Published:** 2024-10-07

**Authors:** Márcia Bastos Convento, Andreia Silva de Oliveira, Mirian Aparecida Boim, Fernanda Teixeira Borges

**Affiliations:** 1 Nephrology Division, Department of Medicine, Federal University of Sao Paulo, Sao Paulo 04038-901, Brazil; andreiaoliveira9921@gmail.com (A.S.d.O.) maboim@unifesp.br (M.A.B.); ft.borges@unifesp.br (F.T.B.); 2 Interdisciplinary Postgraduate Program in Health Sciences, Cruzeiro do Sul University, Sao Paulo 01506-000, Brazil

**Keywords:** umbilical cord mesenchymal stem cell-derived extracellular vesicles, miRNA-126 and anti-miRNA-126, natural nanocarriers

## Abstract

Umbilical cord mesenchymal stem cell-derived extracellular vesicles (UC-EVs) are valuable in nanomedicine as natural nanocarriers, carrying information molecules from their parent cells and fusing with targeted cells. miRNA-126, specific to endothelial cells and derived from these vesicles, supports vascular integrity and angiogenesis and has protective effects in kidney diseases. Objective: This study investigates the delivery of miRNA-126 and anti-miRNA-126 via UC-EVs as natural nanocarriers for treating nephrotoxic injury in vitro. Method: The umbilical cord-derived mesenchymal stem cell and UC-EVs were characterized according to specific guidelines. Rat kidney proximal tubular epithelial cells (tubular cells) were exposed to nephrotoxic injury through of gentamicin and simultaneously treated with UC-EVs carrying miRNA-126 or anti-miRNA-126. Specific molecules that manage cell cycle progression, proliferation cell assays, and newly synthesized DNA and DNA damage markers were evaluated. Results: We observed significant increases in the expression of cell cycle markers, including PCNA, p53, and p21, indicating a positive cell cycle regulation with newly synthesized DNA via BrDU. The treatments reduced the expression of DNA damage marker, such as H2Ax, suggesting a lower rate of cellular damage. Conclusions: The UC-EVs, acting as natural nanocarriers of miRNA-126 and anti-miRNA-126, offer nephroprotective effects in vitro. Additionally, other components in UC-EVs, such as proteins, lipids, and various RNAs, might also contribute to these effects.

## 1. Introduction

Mesenchymal stem cells (MSCs) are multipotent progenitor cells that can migrate to sites of injury and exert immunomodulatory, tissue repair, and regenerative functions. Regenerative medicine has experienced rapid growth, with umbilical cord-derived mesenchymal stem cells (UC-MSCs) showing a notable capacity for differentiating into diverse cell types and possessing a higher proliferation rate for self-renewal, along with lower human leukocyte antigen expression compared to other MSCs [1].

The therapeutic effects of UC-MSCs are mainly due to their paracrine actions, wherein their secretome plays a significant role [2]. Up to 80% of the benefits derived from these cells are estimated to be attributed to paracrine mechanisms [3]. The UC-MSC secretome is composed of a soluble fraction containing cytokines, immunomodulatory molecules, chemokines, and growth factors, and an insoluble extracellular vesicular fraction, which includes various types of extracellular vesicles (EVs) [4].

EVs are nanovesicles essential for cell-to-cell communication, transporting proteins, miRNAs, and anti-miRNAs. They have gained significant attention as novel gene delivery nanocarriers due to their ability to cross biological barriers, reduced toxicity, evasion of the mononuclear phagocytic system, and superior therapeutic index compared to synthetic nanoparticles [5,6,7].

MiRNA-126, predominantly expressed in endothelial cells, plays a crucial role in maintaining vascular integrity and promoting angiogenesis [8]. In the context of acute kidney injury (AKI), damage to the proximal tubules and dysfunction of the renal microvasculature create a detrimental cycle that can lead to the progression of AKI to chronic kidney disease (CKD) [9].

Gentamicin-induced nephrotoxicity, a standard model of AKI, occurs due to the accumulation of the antibiotic in proximal tubular cells, leading to excessive production of reactive oxygen species. This results in oxidative stress, mitochondrial dysfunction, apoptosis, and cellular necrosis. Additionally, gentamicin disrupts the autophagy process, causing the accumulation of damaged organelles, which exacerbates cellular injury and contributes to progressive renal dysfunction. These mechanisms are also seen in other forms of AKI and CKD, where oxidative stress and mitochondrial dysfunction play critical roles in the progression of cellular damage [10].

In clinical studies, miRNA-126 shows varying expression patterns depending on the disease type. Decreased levels of miRNA-126 have been observed in patients with CKD and Diabetes Mellitus when measured in serum and whole blood [11,12]. These lower levels are associated with the prediction and severity of AKI in ICU patients [13,14]. In contrast, elevated miRNA-126 levels have been detected in cases of diabetic nephropathy [15].

miRNA-126 is a post-transcriptional regulator, modulating genes involved in cell proliferation, differentiation, and response to oxidative stress. It targets upstream regulators of TLR signaling, such as Tsc1, a known inhibitor of the mTOR pathway [16], and inhibition of this pathway, as demonstrated by Wen et al. [17], protects against gentamicin-induced nephrotoxicity by reducing cell death and promoting renal regeneration. Bijkerk et al. [18] reported that overexpression of miRNA-126 in the hematopoietic compartment mobilizes vasculogenic progenitor cells through stromal cell-derived factor/CXCR4 signaling, enhancing vascular integrity and supporting renal recovery. Furthermore, Wang et al. [19] found that miRNA-126 reduces inflammation and apoptosis in HUVECs by targeting TRAF7, thereby protecting cells from oxidative stress. Additionally, miRNA-126 delivered via extracellular vesicles (EVs) from endothelial progenitor cells has been shown to protect the kidney from acute ischemic injury by reprogramming hypoxic resident renal cells into a regenerative state [20]. Reis et al. [16] demonstrated that miRNA-126 is among the top 10 enriched miRNAs in MSCs-derived EVs.

A microarray analysis conducted by Jason et al. [21] revealed that the absence of miRNA-126 in endothelial cells can derepress certain genes, some of which have been shown to play a protective role in nephrotoxic models. For instance, Li et al. [22] and Chiba et al. [23] demonstrated that Sirt5 exerts protective effects against cisplatin-induced nephrotoxicity by reducing apoptosis and mitochondrial damage in tubular cells. Satoh et al. [24] highlighted the role of HSP90A in promoting the regeneration of damaged cells following tubular renal injury.

Thus, the main objective of this study was to evaluate, in vitro, the potential of extracellular vesicles derived from umbilical cord mesenchymal stem cells (UC-EVs) as nanocarriers for the efficient delivery of miRNA-126 and anti-miRNA-126 to tubular cells in a nephrotoxic model, investigating whether this treatment could modify cell behavior and promote nephroprotective effects.

## 2. Materials and Methods

### 2.1. In Vivo: Obtaining the Umbilical Cord-Derived Mesenchymal Stem Cell (UC-MSCs)

Animal Model and Ethical Approval: Female Wistar rats were sourced from CEDEME/UNIFESP and maintained under controlled conditions (25 °C/77 °F, alternating light-dark cycles) with unrestricted access to water and chow (Nuvilab, Colombo, PR, Brazil). The study was approved by the Ethical Committee for Experimental Animals at the Federal University of São Paulo, SP, Brazil (approval number 7449230719) and was conducted in accordance with Brazilian guidelines for the care and use of animals in research [25].

Isolation of UC-MSCs: Following an eight-day acclimatization period, rats were assigned to the control group. Umbilical cords were collected from pregnant Wistar rats (*n* = 3) at 21 days of gestation. The fetuses were anesthetized and euthanized by decapitation. The rats were euthanized using an intraperitoneal injection of a lethal dose of 30 mg/kg xylazine and 270 mg/kg ketamine (Agribands do Brasil Ltda., São Paulo, Brazil).

### 2.2. In Vitro Analyses: Umbilical Cord-Derived Mesenchymal Stem Cell (UC-MSCs)

Culture Conditions: UC-MSCs were cultured in DMEM: Dulbecco’s Modified Eagle Medium (DMEM; Sigma-Aldrich, St. Louis, MO, USA) with the addition of 20% FBS: fetal bovine serum (Gibco, Bristol, RI, USA). Cells from passages 4-6 were used for experiments (*N* = 15 for each group). Flow cytometric analysis was performed to confirm the presence of anti-CD45, anti-CD90 markers (both:1:10, BD Biosciences, Franklin Lakes, NJ, USA) and beta-actin (β-actin, 1:1000, Abcam, Cambridge, UK).

Differentiation Potential: The ability of UC-MSCs to differentiate into osteocytes and adipocytes was assessed. UC-MSCs (7 × 10^5^ cells) were grown in specific differentiation media for each cell type, StemPro Osteogenesis and Adipogenesis Differentiation Medium (Gibco, Thermo Fisher, Waltham, MA, USA), at 37 °C with 5% CO_2_, following the manufacturer’s instructions. Media were changed every 3–5 days. After differentiation, cells were stained with 2% Alizarin Red S for osteogenic differentiation and 6.3% Oil Red O for adipogenic differentiation. Nikon microscopy (Leica Microsystems, Wetzlar, Germany) captured and displayed the results through photomicrographs.

Cell proliferation was assessed using the 3-(4,5-dimethylthiazol-2-yl)-2,5-diphenyltetrazolium bromide (MTT) assay between 24–96 h, with results spectrophotometrically measured as described previously [26] and expressed as optical density (OD).

### 2.3. In Vitro Analyses: Umbilical Cord Mesenchymal Stem Cell-Derived Extracellular Vesicles (UC-EVs)

Isolation and Characterization of UC-EVs: The extracellular vesicles were isolated as previously described [27]. Western blotting was conducted as described previously [28]. The protein concentration was confirmed using the Lowry method [29]. Cells were lysed with radioimmunoprecipitation assay buffer centrifuged at 12,000× *g* for 5 min at 4 °C. Proteins were then separated via polyacrylamide gel electrophoresis and transferred to polyvinylidene fluoride membranes. Nonspecific binding sites were blocked using a TBS buffer with 5% albumin. Immunoblots were incubated overnight at 4 °C with antibodies against CD63 and CD81 (both 1:300, Santa Cruz Biotechnology, Dallas, TX, USA). Band intensities were quantified using ImageJ software (version 1.53k).

Transmission Electron Microscopy (TEM): 8 µL drops of UC-EVs in PBS were placed on activated formvar carbon-coated grids (Pyser-SGI Limited, Warwickshire, UK) at room temperature for 5–15 min. At room temperature, UC-EVs were stained with 2% ammonium molybdate for 20 s. Imaging was performed using a Morgagni 268D transmission electron microscope at various magnifications and a voltage of 70 kV, resulting in the export of 16-bit grayscale TIFF images.

Nanoparticle Tracking Analysis (NTA): UC-EVs samples were resuspended in PBS and analyzed using a Malvern NanoSight NS300 (NanoSight, Malvern, UK) to measure their concentration. The process involved recording five 30-s videos to determine particle size and distribution, with results expressed in particles/mL. Protein concentration in the UC-EVs pellet was assessed using the Lowry method [29], indirectly inferring EVs concentration. The treatment involved administering 50 μg/mL of UC-EVs, carrying miRNA-126 or anti-miRNA-126 sequences in the tubular cells.

miRNA Extraction: miRNA was extracted from UC-MSCs and UC-EVs using the mirVana RNA Isolation Kit (Ambion, Thermo Fisher, Waltham, MA, USA), following the manufacturer’s guidelines. RNA quantity was determined spectrophotometrically using a Nanodrop ND-1000. The expression levels of miRNA-126-5p were measured using the MicroRNA TaqMan assay kit (Ambion Thermo Fisher) for microRNAs through real-time quantitative polymerase chain reaction (qRT-PCR) on a GeneAmp Sequence Detection System model ABI Prism 7700 (Applied Biosystems, Waltham, MA, USA). The results were calculated using the 2 (-Delta Delta C(T)) method [30], with expression levels normalized to U6 snRNA and presented in arbitrary units (AU).

Gene silencing: It was reached by specific anti-miRNA inhibitor transfection. The RNAiFect reagent (Qiagen, Germantown, MD, USA) was used to deliver antimir-126-5p (Ambion Thermo Fisher, Waltham, MA, USA) into UC-EVs, with transfection efficiency aimed at reaching at least 80% of cells, determined through reverse transcription PCR. The transfection followed the manufacturer’s protocol and was evaluated by microRNA 126-5p expression through Taqman microRNA assay, and the results were expressed in arbitrary units (AU).

### 2.4. In Vitro Analyses: Tubular Cells Exposed to Nephrotoxic Injury and Simultaneously Treated with UC-EVs Carrying miRNA-126 or Anti-miRNA-126 Sequences

Cell Culture, Nephrotoxic Injury, and Treatments: Proximal tubular epithelial cells from rat kidneys (RPTEC) obtained from the American Type Culture Collection were cultured in F12 medium (Sigma-Aldrich, St. Louis, MO, USA) supplemented with 10% fetal bovine serum (FBS) until they reached confluence. Nephrotoxicity was induced using 2 mM gentamicin (Sigma-Aldrich, St. Louis, MO, USA) for 24 h.

Tubular cells were exposed to nephrotoxic injury and simultaneously treated with 50 μg/mL of UC-EVs carrying miRNA-126: UC-EVs and anti-miRNA-126 sequences: UC-EVs (anti-miRNA-126) at 24 h. This procedure was replicated three times across different periods to ensure consistency and reliability of the experimental outcomes (*n* = 15 for each experimental group).

Experimental Groups: RPTEC were allocated into five groups: the control group (R); RPTEC stimulated with gentamicin (R+G); RPTEC stimulated with gentamicin and treated simultaneously with UC-EVs carrying miRNA-126 sequences [R+G(UC-EV)]; RPTEC stimulated with gentamicin and simultaneously treated with UC-EVs carrying anti-miRNA-126 sequences [R+G(UC-EV)-126], and RPTEC treated with UC-EVs carrying anti-miRNA-126 sequences without gentamicin stimulation [R(UC-EV)-126].

Immunofluorescence was performed as previously described [28]. It was carried out using primary antibodies at a ratio of 1:50 for bromodeoxyuridine (BrdU, Sigma-Aldrich, St. Louis, MO, USA), Ki67, and gamma-H2AX (both from Abcam, Cambridge, MA, USA). TRITC-labeled secondary IgG (1:100, Santa Cruz Biotechnology, Dallas, TX, USA) and 4′,6-diamidino-2-phenylindole (DAPI) staining were applied. Nikon microscopy (Leica Microsystems, Wetzlar, Germany) captured and displayed the results through photomicrographs. ImageJ software quantified the microscopic images, expressing results as fluorescence intensity (%).

Western blotting was performed following previously established protocols [28], and were incubated overnight at 4 °C with antibodies against transformation-related protein 53 (p53, 1:1000, Santa Cruz Biotechnology, Dallas, TX, USA), cyclin-dependent kinase inhibitor 1A (p21, 1:500, Sigma-Aldrich, St. Louis, MO, USA), proliferating cell nuclear antigen (PCNA, 1:1000, Santa Cruz Biotechnology, Dallas, TX, USA), c-Myc (1:500, Sigma-Aldrich, St. Louis, MO, USA), beta-actin (β-actin, 1:1000, Abcam, Cambridge, UK), and glyceraldehyde-3-phosphate dehydrogenase (GAPDH, 1:1000, Santa Cruz Biotechnology, Dallas, TX, USA). Band intensities were quantified using ImageJ, expressed as the ratio to β-actin or GAPDH.

Cell proliferation was assessed using the MTT assay at 24–72 h.

### 2.5. Statistical Analyses

Data analysis was carried out using Action Stat software (version 3.3.2) for Windows, beginning with the Shapiro–Wilk test to assess normality. For non-normally distributed data, the Kruskal–Wallis test was applied, followed by the Bonferroni correction. For normally distributed data, comparisons were made using the Tukey post-hoc test. Results are expressed as mean ± standard error, with statistical significance set at *p* < 0.05.

## 3. Results

### 3.1. Umbilical Cord-Derived Mesenchymal Stem Cells (UC-MSCs)

**Figure 1:**
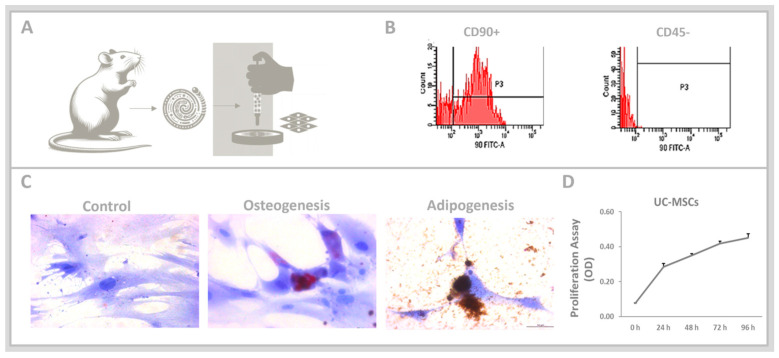
(**A**). Umbilical cords were removed from pregnant female Wistar, and UC-MSCs were used. (**B**). Flow cytometry analysis of UC-MSCs was carried out using FITC-conjugated antibodies (red histograms) to identify cell surface markers (CD45− and CD90+). (**C**). The cells demonstrated multipotency, which is characteristic of UC-MSCs, with the ability to differentiate into osteogenic and adipogenic lineages. (**D**). Cell proliferation was assessed using the MTT assay. Data are reported as mean ± standard error of the mean.

Umbilical cords were removed from pregnant female Wistar, and UC-MSCs were used, as depicted in Figure 1A. The immunophenotyping conducted via flow cytometric analysis, shown in Figure 1B, indicated that the cells were negative for CD45 but positive for CD90, as expected. Following a 21-day induction period, the UC-MSCs showed potential differentiation into osteocytes and adipocytes (Figure 1C). The MTT assay confirmed the increased rate of proliferation in the UC-MSCs between 24 and 96 h (Figure 1D).

### 3.2. Umbilical Cord Mesenchymal Stem Cell-Derived Extracellular Vesicles (UC-EVs)

**Figure 2:**
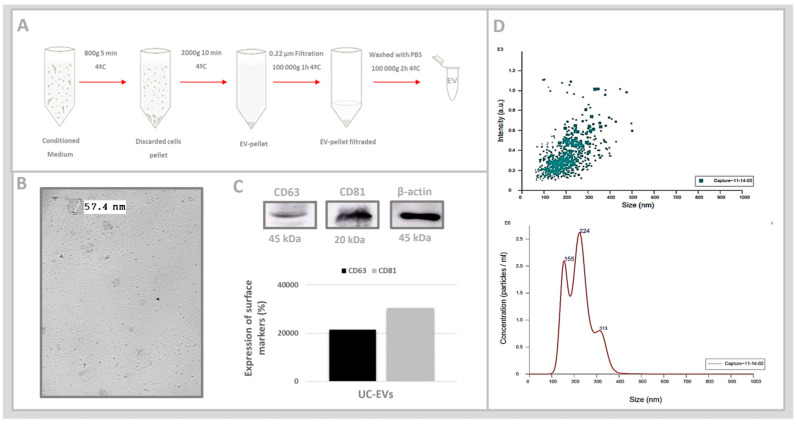
(**A**). UC-EVs isolation through differential ultracentrifugation. The secretome was first centrifuged at 800× *g* for 10 min to remove suspended cells, followed by 10-min centrifugation at 2000× *g* to eliminate cell debris. It was then filtered through a 0.22 μm syringe filter and ultracentrifuged at 100,000× *g* for 2 h to collect UC-EVs as a pellet. The pellet was washed with cold 1× PBS and centrifuged again at 100,000× *g* for 2 h to eliminate protein aggregates and free proteins. (**B**). Visualization of UC-EVs using transmission electron microscopy (TEM) with a filter set to focus on UC-EVs around 50 nm. (**C**). Western blotting images were quantitatively analyzed using ImageJ, demonstrating the expression of EV-surface markers CD63 and CD81. (**D**). Particle sizes and relative scattering intensity of UC-EV samples were determined using nanoparticle tracking analysis (NTA). Data are reported as mean ± standard error of the mean.

UC-EVs were isolated from the secretome of UC-MSCs through differential ultracentrifugation (Figure 2A). TEM imaging confirmed the morphology of extracellular vesicles as double-layered, cup-shaped membrane structures with diameters around 50 nm (Figure 2B). Immunoblotting analyses were performed to assess the presence of the extracellular vesicle (EV) surface markers CD81 and CD63, transmembrane tetraspanin proteins commonly found in UC-EVs. The presence of these markers in UC-EVs was confirmed by visualizing the bands corresponding to CD63 and CD81 on membranes following the Western blotting assay. The associated graphical quantifications, normalized to the reference protein β-actin, showed consistent expression levels of both markers, validating the identity of the UC-EVs (Figure 2C). The analysis of UC-EVs by nanoparticle tracking analysis (NTA) identified two distinct populations, with average sizes of 156 nm and 223.6 nm, classified as exosomes and microvesicles, respectively. Most data points were clustered between 50 and 300 nm based on size and relative scattering intensity. This range aligns with the typical size of extracellular vesicles, such as exosomes (30–150 nm) and microvesicles (100–1000 nm), confirming the identity of UC-EVs (Figure 2D).

**Figure 3:**
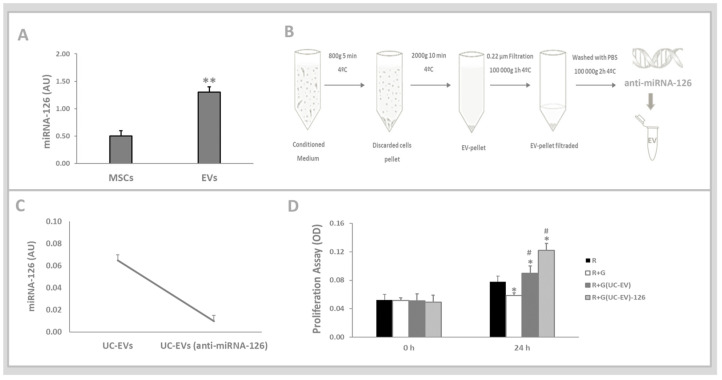
Umbilical cord mesenchymal stem cell-derived extracellular vesicles (UC-EVs) carrying miRNA-126 or anti-miRNA-126 sequences. (**A**). Quantitative polymerase chain reaction analysis showing miRNA-126. (**B**). UC-EVs isolation through differential ultracentrifugation. (**B**). UV-EV isolation through differential ultracentrifugation. The secretome was first centrifuged at 800× *g* for 10 min to remove suspended cells, followed by 10-min centrifugation at 2000× *g* to eliminate cell debris. It was then filtered through a 0.22 μm syringe filter and ultracentrifuged at 100,000× *g* for 2 h to collect UC-EVs as a pellet. The pellet was washed with cold 1× PBS and centrifuged again at 100,000× *g* for 2 h to eliminate protein aggregates and free proteins. The anti-miRNA-126 was transfected according to the manufacturer’s instructions. (**C**). Quantitative analysis of miRNA-126 expression using polymerase chain reaction (qPCR). (**D**). At 24 h, tubular cell proliferation was evaluated using the MTT assay. Data are presented as mean ± standard error of the mean (SEM). A significance level of 5% (*p* < 0.05) was used for testing the null hypothesis. An asterisk (**) indicates a comparison with the UC-MSCs group, (*) indicates a comparison with the R group (control), while a hash (#) indicates a comparison with the R+G group (the tubular cells exposed to nephrotoxic injury).

Quantitative PCR analysis confirmed the presence of miRNA-126 in both UC-EVs and UC-MSCs, with a greater abundance in UC-EVs compared to the parent cells (Figure 3A). Figure 3B shows UC-EVs isolation through the differential ultracentrifugation method by adding the anti-miRNA-126 oligonucleotides. Figure 3C shows the effectiveness of anti-miRNA-126 delivery in the UC-EVs, which validated the transfection quality. Figure 3D illustrates that gentamicin-stimulated tubular cells simultaneously treated with UC-EVs carrying either miRNA-126 or anti-miRNA-126 showed a significantly increased proliferative capacity at 24 h compared to both the control group and the gentamicin-exposed tubular cells that were not treated.

### 3.3. Tubular Cells Exposed to Nephrotoxic Injury and Simultaneously Treated with UC-EVs Carrying miRNA-126 Sequences at 24 h

**Figure 4:**
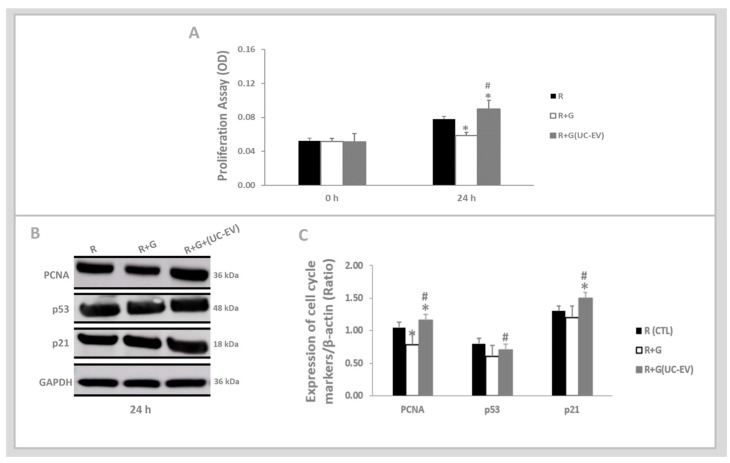
Tubular cells exposed to nephrotoxic injury and simultaneously treated with UC-EVs carrying miRNA-126 sequences. (**A**). At 24 h, tubular cell proliferation was evaluated using the MTT assay. (**B**,**C**). The expression levels of the cell cycle markers, p53, p21, and PCNA, were quantitatively assessed from Western blot images using ImageJ software. Data are presented as mean ± standard error of the mean (SEM). A significance level of 5% (*p* < 0.05) was used for testing the null hypothesis. An asterisk (*) indicates a comparison with the R group (control), while a hash (#) indicates a comparison with the R+G group (the tubular cells exposed to nephrotoxic injury).

The MTT assay, as shown in Figure 4A, indicates a pronounced decline in the proliferative rate of tubular cells stimulated with gentamicin (R+G) compared to the control condition (R). Notably, at 24 h, tubular cells exposed to gentamicin and simultaneously treated with UC-EVs carrying miRNA-126 exhibited a significant enhancement in proliferative capacity, surpassing both the control group and the untreated gentamicin-stimulated tubular cells.

Figure 4B presents the analysis of p21, p53, and PCNA cell cycle markers at 24 h, coupled with their corresponding graphical quantifications in Figure 4C. The expression levels of PCNA were lower in gentamicin-stimulated tubular cells compared to the control group. In contrast, the expression levels of p53 and p21 in gentamicin-stimulated tubular cells were similar to those observed in the control group.

Gentamicin-stimulated tubular cells simultaneously treated with UC-EVs carrying miRNA-126 displayed a markedly higher increase in PCNA and p21 levels compared to both the control group and the untreated gentamicin-stimulated tubular cells. Additionally, this group showed a more pronounced elevation in p53 levels compared to the untreated gentamicin-stimulated tubular cells.

### 3.4. Tubular Cells Exposed to Nephrotoxic Injury and Simultaneously Treated with UC-EVs Carrying Anti-miRNA-126 Sequences at 24 h

**Figure 5:**
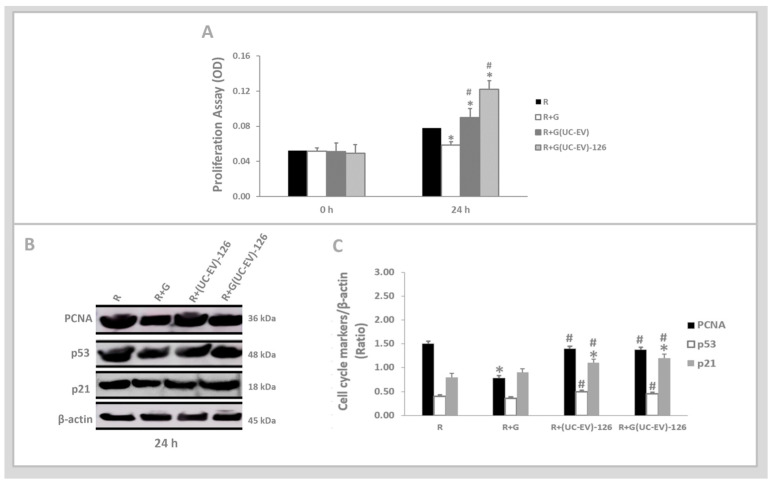
Tubular cells exposed to nephrotoxic and anti-miRNA-126 sequences. (**A**). At 24 h, tubular cell proliferation was evaluated using the MTT assay. (**B**,**C**). The expression levels of the cell cycle markers, p53, p21, and PCNA, were quantitatively assessed from Western blot images using ImageJ software. Data are presented as mean ± standard error of the mean (SEM). A significance level of 5% (*p* < 0.05) was used for testing the null hypothesis. An asterisk (*) indicates a comparison with the R group (control), while a hash (#) indicates a comparison with the R+G group (the tubular cells exposed to nephrotoxic injury).

The MTT assay, as shown in Figure 5A, indicates a pronounced decline in the proliferative rate of tubular cells stimulated with gentamicin (R+G) compared to those in the control condition (R). Gentamicin-stimulated tubular cells simultaneously treated with UC-EVs carrying anti-miRNA-126 exhibited a significant increase in proliferative capacity at 24 h, outperforming both the control group and the tubular cells stimulated with gentamicin untreated.

Figure 5B shows the analysis results of the p53, PCNA, and p21 cell cycle markers at 24 h, coupled with their corresponding graphical quantifications in Figure 5C. The expression levels of PCNA were significantly lower in the gentamicin-stimulated tubular cells compared to the control group. The p21 and p53 showed similar levels in the tubular cells stimulated with gentamicin compared to the control group.

Gentamicin-stimulated tubular cells simultaneously treated with UC-EVs carrying anti-miRNA-126 displayed a markedly higher increase in PCNA and p53 levels than those stimulated with gentamicin without treatment. Furthermore, this group demonstrated a significantly higher increase in p21 levels relative to the control group and the tubular cells stimulated with gentamicin without treatment.

### 3.5. Tubular Cells Exposed to Nephrotoxic Injury and Simultaneously Treated with UC-EVs Carrying miRNA 126 or Anti-miRNA-126 Sequences at 72 h

**Figure 6:**
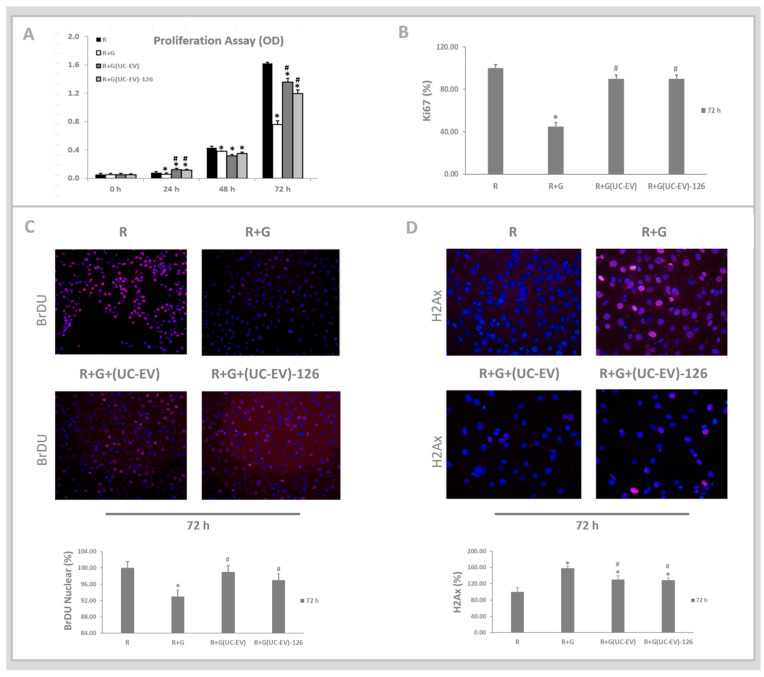
Tubular cells exposed to nephrotoxic injury and simultaneously treated with UC-EVs carrying miRNA-126 or anti-miRNA-126 sequences at 72 h. (**A**). At 24–72 h, tubular cell proliferation was evaluated using the MTT assay. (**B**). At 72 h, tubular cell proliferation was assessed using the Ki-67 marker. (**C**). BrdU immunofluorescence assay: the fraction of cells that incorporated BrdU into their DNA (TRITC), whose nuclei were stained with DAPI, and microscopic images were quantified with the ImageJ software. (**D**). H2Ax immunofluorescence assay: the cell fraction illustrates the distribution of γ-H2AX labeling (TRITC), whose nuclei were stained with DAPI, and microscopic images were quantified with the ImageJ software. Scale bar = 100 μm. Data are presented as mean ± standard error of the mean (SEM). A significance level of 5% (*p* < 0.05) was used for testing the null hypothesis. An asterisk (*) indicates a comparison with the R group (control), while a hash (#) indicates a comparison with the R+G group (the tubular cells exposed to nephrotoxic injury).

The impact of gentamicin-stimulated tubular cells simultaneously treated with UC-EVs carrying miRNA-126 or anti-miRNA-126 was assessed at 72 h. As shown in Figure 6A, the MTT assay revealed a significant reduction in the proliferative capacity of the tubular cells stimulated with gentamicin (R+G) compared to those in the control group (R). Tubular cells exposed to nephrotoxic injury and simultaneously treated with UC-EVs, whether carrying miRNA-126 or anti-miRNA-126, exhibited significantly enhanced proliferation at 72 h compared to the tubular cells stimulated with gentamicin without treatment.

Figure 6B shows that the Ki67 marker, which indicates cell proliferation, significantly reduced expression in the gentamicin-stimulated tubular cells (R+G) compared to those in the control condition (R). However, tubular cells exposed to nephrotoxic injury and simultaneously treated with UC-EVs, regardless of whether they were carrying miRNA-126 or anti-miRNA-126, exhibited significantly enhanced Ki67 expression at 72 h compared to the tubular cells stimulated with gentamicin without treatment, as depicted in Figure 6B.

Figure 6C showed the protein synthesis results via an immunofluorescence assay, coupled with their corresponding graphical quantifications for BrDU, which indicated newly synthesized DNA, and H2Ax, which indicated DNA damage. There was a decrease in the BrDU expression in the tubular cells stimulated with gentamicin (R+G) compared to those in the control condition (R). However, gentamicin-stimulated tubular cells and simultaneously treated with UC-EVs, regardless of whether they were carrying miRNA-126 or anti-miRNA-126, exhibited significantly enhanced BrDU expression, indicating newly synthesized DNA at 72 h, compared to the tubular cells stimulated with gentamicin without treatment, as shown in Figure 6C.

Figure 6D shows the protein synthesis results via an immunofluorescence assay and their corresponding graphical quantifications for H2Ax. The results demonstrated a significant increase in the H2Ax expression in the tubular cells stimulated with gentamicin (R+G) compared to those in the control condition (R). However, tubular cells exposed to nephrotoxic injury and simultaneously treated with UC-EVs, regardless of whether they were carrying miRNA-126 or anti-miRNA-126, exhibited significantly decreased in the H2Ax expression, indicating less DNA damage at 72 h, compared to the tubular cells stimulated with gentamicin without treatment.

**Figure 7:**
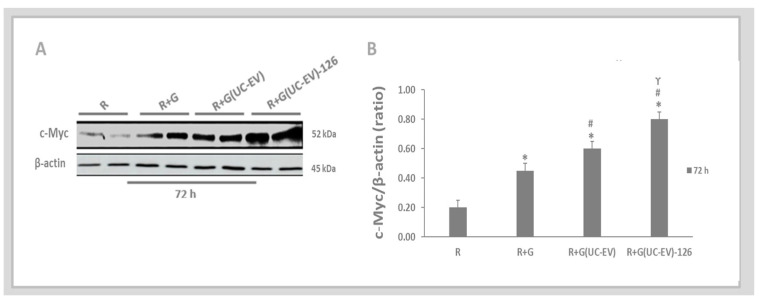
The paracrine mechanism of extracellular vesicles derived from umbilical cord mesenchymal stem cells (UC-EVs) involves their ability to transfer molecules (miRNA-126 and anti-miRNA-126) to target tubular cells. (**A**,**B**). The expression levels of the c-Myc were quantitatively assessed from Western blot images using ImageJ software. Data are presented as mean ± standard error of the mean (SEM). A significance level of 5% (*p* < 0.05) was used for testing the null hypothesis. An asterisk (*) indicates a comparison with the R group (control), while a hash (#) indicates a comparison with the R+G group (the tubular cells exposed to nephrotoxic injury), and gamma (ϒ) compared with the group of tubular cells exposed to nephrotoxic injury and simultaneously treated with UC-EVs carrying anti-miRNA-126 versus the group of tubular cells exposed to nephrotoxic injury and simultaneously treated with UC-EVs carrying miRNA-126.

The paracrine mechanism of extracellular vesicles derived from umbilical cord mesenchymal stem cells (UC-EVs) involves their ability to transfer molecules (miRNA-126 and anti-miRNA-126) to target tubular cells. Figure 7A,B showed an increase in c-Myc expression in the tubular cells stimulated with gentamicin (R+G) compared to the control group (R). However, gentamicin-stimulated tubular cells and simultaneously treated with umbilical cord mesenchymal stem cells, containing either miRNA-126 or anti-miRNA-126, displayed a significantly greater rise in c-Myc expression when compared to both the control situation and gentamicin-stimulated tubular cells that were not treated. Lower levels of miRNA-126 remove the inhibition of the 3′-UTR regions of c-Myc mRNA. This lack of inhibition facilitates an increase in the translation process, leading to higher levels of c-Myc protein [31,32]. Thus, the comparison between UC-EVs treatments revealed that tubular cells exposed to nephrotoxic injury and simultaneously treated with UC-EVs carrying anti-miRNA-126 exhibited a significant increase in c-Myc expression compared to those treated with UC-EVs carrying miRNA-126.

## 4. Discussion

UC-MSCs and UC-EVs were characterized following the guidelines of the International Society for Cellular Therapy and MISEV2018, respectively, confirming the notable proliferative capacity of the UC-MSCs and the enrichment of pro-angiogenic miRNA-126 in the UC-EVs compared to their originating cells [33,34]. The literature corroborates that specific miRNAs are expressed at higher levels in extracellular vesicles than in their donor cells [35,36].

Apoptosis in the tubular cells is primarily due to the processing of DNA double-strand breaks (DSBs) caused by gentamicin [37,38]. Apoptosis is triggered when PCNA is non-functional, absent, or expressed in lower quantities [39]. Compared to the tubular cells, the tubular cells exposed to nephrotoxic injury showed a decrease in proliferative rate and levels of the cell proliferation marker, PCNA.

Kidney cell turnover is minimal under normal conditions, with tubular cells mainly in the G0 quiescent or G1 phase for growth [40]. After irreversible injury, tubular cells arrest in the G2 phase to check for replication errors, leading to maladaptive repair [41]. Conversely, after reversible injury, surviving tubular cells re-enter the cell cycle to replace lost cells, with about two-thirds successfully progressing through the DNA duplication S phase [42,43,44]. These processes are meticulously regulated by specific molecules managing cell cycle progression through various checkpoints.

In the tubular cells exposed to nephrotoxic injury and simultaneously treated with UC-EVs carrying miRNA-126 or anti-miRNA-126, there was the replacement of lost tubular cells with new ones evidenced by an increased proliferation rate and elevated PCNA expression at 24 h. PCNA is a vital protein influencing cell life and death decisions, which are also induced by p53 [39]. When PCNA levels are high in the presence of p53, it leads to DNA repair [39,45,46]. As the guardian of the genome, p53 monitors the genome for signs of DNA damage, such as double-strand breaks (DSBs) caused by gentamicin [47,48].

The p53 up-regulation can halt cell cycle progression at the G2 phase to check for replication errors or induce G1 phase arrest via p21 induction, facilitating DNA damage repair [46,47,48,49]. The expression of p21 protects tubular cells from apoptosis by providing additional time for DNA repair and preventing premature progression towards cell death [49,50,51]. This protective mechanism is mainly observed when tubular cells, exposed to nephrotoxic injury, re-enter the cell cycle, demonstrating a crucial role of p21 in cellular defense [50,51].

In the tubular cells exposed to nephrotoxic injury and simultaneously treated with UC-EVs carrying miRNA-126 or anti-miRNA-126 sequences, there was an increase in the expression levels of PCNA, p53, and the checkpoint protein p21. Together, PCNA, p53, and p21 ensure the accurate completion of each cell cycle phase before allowing progression to the next, playing critical roles in cellular repair and regulation mechanisms post-injury.

This study assessed cell behavior after 24 h post-treatment, as only cells with intact DNA can advance beyond the checkpoint into the next cell cycle phase. In the tubular cells exposed to nephrotoxic injury and simultaneously treated with UC-EVs carrying miRNA-126 or anti-miRNA-126, a pattern was observed: an increase in proliferation at 24 h, a decrease at 48 h, and another increase at 72 h. This result suggests a temporary cell cycle arrest at 48 h for DNA repair once cell proliferation resumed at 72 h. The increased expression of BrdU, a marker of newly synthesized DNA [52], indicated the presence of newly synthesized DNA. Additionally, the reduction in the phosphorylation of histone H2Ax-positive cells showed fewer cells with DNA damage at 72 h, as phosphorylation of histone H2Ax is a sensor for double-strand breaks (DSBs) caused by gentamicin [53].

The paracrine mechanism of UC-EVs allows for the direct transfer of molecules (miRNA-126 and anti-miRNA-126) to target tubular cells, as demonstrated by the increased expression of c-Myc in tubular cells treated with UC-EVs containing anti-miRNA-126. Reduced levels of miRNA-126 remove the inhibition on the 3′-UTR regions of c-Myc mRNA, resulting in enhanced translation and higher levels of c-Myc expression [32]. This confirms that the anti-miRNA oligonucleotides effectively silenced miRNA-126 in UC-EVs, indicating that tubular cells were influenced by this silencing mechanism, which led to increased c-Myc expression. Considering that c-Myc is also involved in key physiological processes such as cell cycle regulation, metabolism, differentiation, proliferation, and angiogenesis [31,54,55,56,57,58], the observed increase in c-Myc may represent an alternative molecular pathway that may have contributed to the nephroprotective effects observed in tubular cells following treatment with UC-EVs without miRNA-126 expression during the study period.

Our study integrates two alignments from the literature: the efficacy of EVs as nanocarriers of miRNA-126 and anti-miRNA-126 [18,19,20] and the context-dependent expression of miRNA-126 in renal protection [11,12,13,14,15]. Furthermore, our results suggest that treatment with UC-EVs containing miRNA-126 played a protective role in tubular cells. It is possible that inhibiting this miRNA with anti-miRNA-126 could lead to the derepression of genes that promote cell survival and proliferation while reducing DNA damage in a nephrotoxicity-induced AKI.

However, beyond miRNA-126, other constituents of UC-EVs, including proteins, lipids, and various RNAs or miRNAs, likely play a role in the nephroprotective effects observed. This aligns with numerous studies employing stem cell-derived extracellular vesicles for kidney disease treatment [59,60,61,62,63], highlighting the intricate interplay of multiple factors within EVs that collectively enhance their properties. The diverse array of molecules within UC-EVs suggests that they deliver a broad spectrum of therapeutic agents that synergistically promote renal recovery and guard against nephrotoxicity. Unfortunately, this study did not thoroughly explore the other molecular pathways activated or inhibited in tubular cells after treatment, leaving its full nephroprotective potential to be elucidated in future research.

We recognize the importance of testing UC-EVs with miRNA-126 and anti-miRNA-126 in animal models better to understand their therapeutic potential and mechanisms in nephrotoxic injury. However, our current study’s limitations prevented a detailed analysis of the effects of different miRNA-126 levels or its absence in tubular cells. Future studies should systematically vary miRNA-126 concentrations in UC-EVs and assess their impact on nephrotoxic models to clarify the dual role of miRNA-126, whether it is dose-dependent, context-specific, or influenced by other factors. This will be crucial for interpreting our findings and developing targeted therapies.

Nevertheless, as a cell-free therapy, using EVs as nanocarriers is promising. EV-based treatments offer significant potential for personalized medicine. For example, Shimbo et al. [64] engineered bone marrow-MSCs-derived extracellular vesicles to increase the expression of miRNA-143, suppressing proliferation, migration, and invasion in osteosarcoma cells. Similarly, Munoz et al. [65] demonstrated that using bone marrow-MSCs-derived extracellular vesicles loaded with anti-miRNA-9 enhanced the sensitivity of chemoresistant glioblastoma multiforme cells to chemotherapy by targeting and reducing miR-9 expression. These studies exemplify the potential of using EVs for targeted delivery of miRNAs and anti-miRNAs to target specific pathways.

The advantage of using mesenchymal stem cell-derived natural extracellular vesicles over synthetic nanocarriers is evident in their efficiency and biocompatibility. Many natural and synthetic nanotransfecting vehicles have been developed to deliver miRNA and anti-miRNA efficiently. However, synthetic vehicles face challenges such as low success rates due to their interactions, stronger RNA binding that reduces delivery rates inside cells, decreased serum half-life due to biodegradability, and increased cytotoxicity [5,6]. An ideal transfection vehicle must balance these factors within a single system.

EVs provide a natural and efficient transport route. They serve as superior miRNA and anti-miRNA delivery mechanisms compared to synthetic carriers due to their balanced properties, improved therapeutic index, and natural tropism with fewer side effects [5,6]. Schindler et al. [7] demonstrated that synthetic nanotransfecting vehicles require a 20- to 80-fold higher concentration to achieve a signal comparable to that produced by EVs. In the future, EV-based drug delivery will emerge as a promising field with the potential to revolutionize therapeutic interventions [66,67,68,69,70,71,72,73].

## 5. Conclusions

Our research revealed a dual role for miRNA-126 in nephrotoxic injuries. Its expression promoted nephroprotective effects, while its inhibition may have activated alternative molecular pathways that increased cell proliferation and DNA repair. Other components within UC-EVs, such as proteins, lipids, and various RNAs or miRNAs, may also have contributed to the observed therapeutic effects. However, the study has certain limitations, as it did not fully explore other molecular pathways activated by miRNA-126 expression or inhibition in tubular cells. Despite these limitations, UC-EVs demonstrated their potential as natural nanocarriers, modulating essential cellular pathways and showing therapeutic potential in gentamicin-induced nephrotoxicity in vitro. Future studies should aim to elucidate these mechanisms further and validate the therapeutic potential of UC-EVs-delivered miRNA-126 and anti-miRNA-126 in various contexts of renal diseases.

## Data Availability

Derived data supporting the findings of this study are available from the corresponding author [M.B.C.] on request.
